# Erratum for Roy et al., “Chikungunya Virus Infection Impairs the Function of Osteogenic Cells”

**DOI:** 10.1128/mSphere.00899-20

**Published:** 2020-09-16

**Authors:** Enakshi Roy, Wen Shi, Bin Duan, St Patrick Reid

**Affiliations:** a Department of Pathology & Microbiology, University of Nebraska Medical Center, Omaha, Nebraska, USA; b Mary & Dick Holland Regenerative Medicine Program, Division of Cardiology, Department of Internal Medicine, University of Nebraska Medical Center, Omaha, Nebraska, USA

## ERRATUM

Volume 5, no. 3, e00347-20, 2020, https://doi.org/10.1128/mSphere.00347-20. In Fig. 5, the graph in panel F was inadvertently duplicated in panel E. The correct graph for panel E is shown in Fig. 5 below.

**FIG 5:**
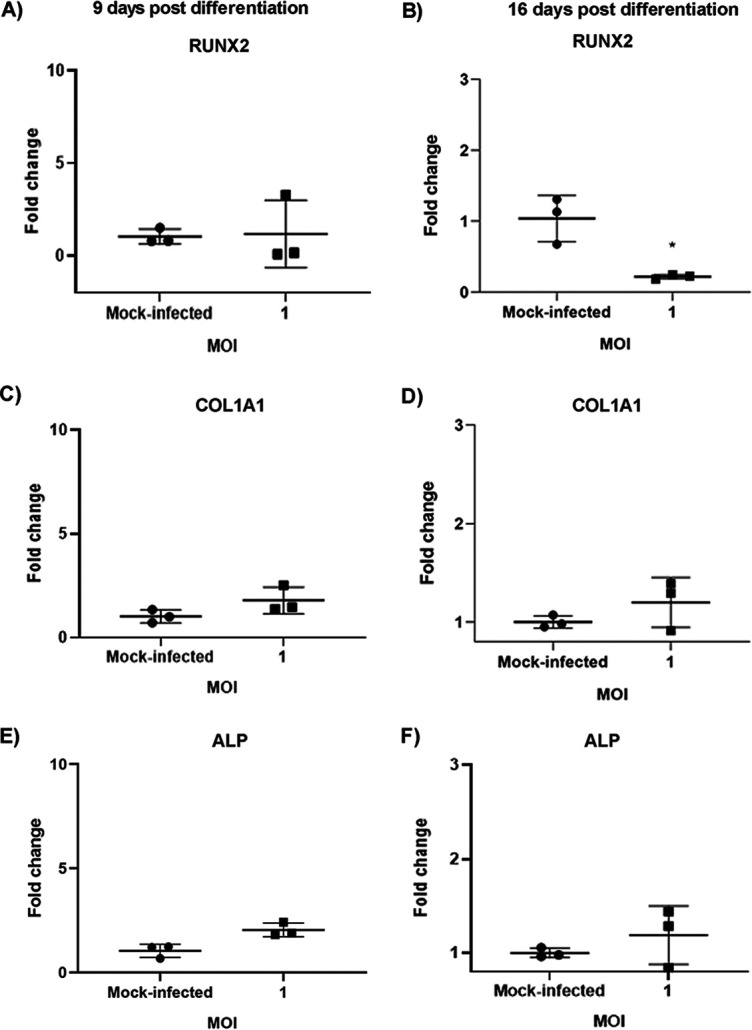
Effect of CHIKV infection on gene expression of osteogenic marker genes. Osteogenic cells at 7 or 14 dpd were mock-infected or infected with CHIKV at MOI = 1. (A to F) Gene expression of the osteogenic marker genes (RUNX2, COL1A1, and ALP) was determined by qRT-PCR at 9 (A, C, E) or 16 dpd (B, D, F) (and at 48 hpi). Graphs show fold change in gene expression. Fold change was calculated by the 2^−ΔΔ^*^CT^* method. Peptidylprolyl isomerase A (PPIA) was used as a housekeeping gene; *n* = 3. Error bars show mean ± SEM. Significant changes are represented as *P* values (*, *P* < 0.05).

